# Protease-Activated Receptor-2 Regulates Neuro-Epidermal Communication in Atopic Dermatitis

**DOI:** 10.3389/fimmu.2020.01740

**Published:** 2020-08-12

**Authors:** Timo Buhl, Akihiko Ikoma, Cordula Kempkes, Ferda Cevikbas, Mathias Sulk, Joerg Buddenkotte, Tasuku Akiyama, Debbie Crumrine, Eric Camerer, Earl Carstens, Michael P. Schön, Peter Elias, Shaun R. Coughlin, Martin Steinhoff

**Affiliations:** ^1^Department of Dermatology and Surgery, University of California, San Francisco, San Francisco, CA, United States; ^2^Department of Dermatology, Venereology and Allergology, University Medical Center Göttingen, Göttingen, Germany; ^3^Department of Dermatology and UCD Charles Institute for Translational Dermatology, University College Dublin, Dublin, Ireland; ^4^Department of Dermatology, University Hospital Münster, Münster, Germany; ^5^Department of Dermatology and Venerology, Hamad Medical Corporation, Doha, Qatar; ^6^Translational Research Institute, Academic Health System, Hamad Medical Corporation, Doha, Qatar; ^7^Department of Dermatology, Anatomy and Cell Biology, Temple Itch Center, Temple University, Philadelphia, PA, United States; ^8^Department of Neurobiology, Physiology and Behavior, University of California, Davis, Davis, CA, United States; ^9^INSERM U970, Paris Cardiovascular Research Centre, Paris, France; ^10^Cardiovascular Research Institute, University of California, San Francisco, San Francisco, CA, United States; ^11^Department of Dermatology, Medical School, University of Qatar, Doha, Qatar; ^12^School of Medicine, Weill Cornell Medicine-Qatar, Doha, Qatar; ^13^Department of Dermatology, Weill Cornell Medicine, New York, NY, United States

**Keywords:** atopic dermatitis, protease-activated receptor-2, PAR2, endothelin, house dust mite, dorsal root ganglion, neuro-immunology

## Abstract

**Background:** Activation of protease-activated receptor-2 (PAR2) has been implicated in inflammation, pruritus, and skin barrier regulation, all characteristics of atopic dermatitis (AD), as well as Netherton syndrome which has similar characteristics. However, understanding the precise role of PAR2 on neuro-immune communication in AD has been hampered by the lack of appropriate animal models.

**Methods:** We used a recently established mouse model with epidermal overexpression of PAR2 (PAR2OE) and littermate WT mice to study the impact of increased PAR2 expression in epidermal cells on spontaneous and house dust mite (HDM)-induced skin inflammation, itch, and barrier dysfunction in AD, *in vivo* and *ex vivo*.

**Results:** PAR2OE newborns displayed no overt abnormalities, but spontaneously developed dry skin, severe pruritus, and eczema. Dermatological, neurophysiological, and immunological analyses revealed the hallmarks of AD-like skin disease. Skin barrier defects were observed before onset of skin lesions. Application of HDM onto PAR2OE mice triggered pruritus and the skin phenotype. PAR2OE mice displayed an increased density of nerve fibers, increased nerve growth factor and endothelin-1 expression levels, alloknesis, enhanced scratching (hyperknesis), and responses of dorsal root ganglion cells to non-histaminergic pruritogens.

**Conclusion:** PAR2 in keratinocytes, activated by exogenous and endogenous proteases, is sufficient to drive barrier dysfunction, inflammation, and pruritus and sensitize skin to the effects of HDM in a mouse model that mimics human AD. PAR2 signaling in keratinocytes appears to be sufficient to drive several levels of neuro-epidermal communication, another feature of human AD.

## Introduction

Protease-activated receptors (PARs) constitute a family of G protein-coupled receptors activated by proteolytic cleavage of their extracellular N-termini. PARs may be activated by various proteases generated by exogenous (e.g., bacteria, mites, plants, and allergens) or endogenous sources (plasma coagulation proteases and proteases from epithelium, endothelium, fibroblasts, or immune cells) (1–3). Possible roles for PAR2 in inflammation and neuro-immune communication in various organs have been described (4–13).

By activation of PAR2 in the skin, proteases such as house dust mite (HDM) allergens, bacterial proteases, kallikreins, matriptase, trypsin-4, or prostasin may contribute to important biological processes including epidermal barrier homeostasis, innate and adaptive immunity, leukocyte recruitment, pigmentation, fibrosis, pruritus, and pain (14–21). Recent studies indicate an important function of PAR2 in atopic dermatitis (AD) and Netherton syndrome. This latter condition is a rare genetic disease caused by mutations in SPINK5, encoding the key serine protease inhibitor LEKTI in the epidermis leading to AD-like skin symptoms (22).

AD is one of the most common chronic inflammatory skin diseases. It is characterized by skin changes such as erythema, edema, and lichenification, in addition to the hallmark symptom of pruritus (itch) (23). Indeed, chronic pruritus affects 87–100% of patients with AD. The inflammatory infiltrate of AD is characterized by excessive T cell activation, specifically TH2, TH17, TH22 cells, in addition to TH1 cells, depending on stage, severity, and disease subtype (24). Cytokines such as IL-4, −13, −31, −22, TARC, and TSLP appear to play an essential role for the accumulation of T cells, macrophages, and mast cells in AD. The cross-communication between immune cells and the epidermis, which is bidirectional, is still a matter of much debate (“inside-out” vs. “outside-in” theory) (25–28). In addition, the links that define AD as an immune disease with a strong neurological association (formerly named “neurodermatitis”) are still poorly understood on the cellular level.

It is generally accepted that exogenous agents such as *S. aureus*, plants, or HDM can act as trigger factors for AD. Several such agents can produce proteases that are capable of PAR2 activation on keratinocytes, thereby inducing skin barrier disruption, cytokine release, or NF-kB activation (29–31). Of note, major HDM allergens have intrinsic protease activity (e.g., Der p1, p3, and p9) that may alter epidermal skin barrier disruption through PAR2 activation, cytokine release, and leukocyte recruitment (4, 32, 33). Epithelial cells can directly promote itch by communication to cutaneous sensory neurons, which cluster their cell bodies in the dorsal root ganglion (DRG). Thus, the protease-keratinocyte-PAR2 axis may be important in the induction phase of AD. By “sensing danger molecules” such as proteases released from environmental or endogenous trigger factors, this axis may result in the induction of eczema-like inflammation and pruritus.

To test this hypothesis, we utilized mice that overexpress PAR2 in keratinocytes (PAR2OE) and stimulated them with house dust mite proteases. Our *in vitro, in vivo* and *ex vivo* studies clearly demonstrate that PAR2OE mice spontaneously develop AD-like dermatitis with characteristic inflammatory infiltrate, increased IgE and severe pruritus. These findings suggest that epidermal PAR2 may function as a “sensor receptor” for environmental proteases and trigger an AD-like phenotype with inflammation and pruritus. Thus, PAR2 antagonism and/or selective protease inhibitors may represent a novel approach for the treatment of AD.

## Materials and Methods

### Nomenclature

PAR2 (a.k.a. F2RL1) and Par2 (a.k.a. F2rl1) refer to human and mouse protease-activated receptor-2 protein, respectively.

### Mice

PAR2 overexpressing mice were generated by inserting a cassette consisting of the mouse Par2 coding sequence followed by an internal ribosomal entry site and the lacZ reporter gene at the start codon of the grainyhead-like-3 (*Grhl3*) gene (*Grhl3*^Par2/+^) by homologous recombination (6). Consistent with simultaneous interruption of the *Grhl3* gene, mice with Par2 inserted in both alleles (*Grhl3*^Par2/Par2^) died perinatally with spina bifida. *Grhl3*^Par2/+^ mice were used throughout this study for transgenic Par2 overexpression. For clarity and readability of the manuscript, we will refer to the *Grhl3*^Par2/+^ as PAR2-overexpressing (PAR2OE) mice. Heterozygous PAR2OE and their littermate wild-type controls (8–12 weeks old) were used for all experiments, unless other ages are indicated. Mice were housed under specific pathogen-free conditions with food and water ad libitum, without antibiotic treatment, and no specific diet. All experiments were approved by the UCSF and UCD-Institutional Animal Care and Use Committee and conducted in accordance with the National Institutes of Health Guide for Care and Use of Laboratory Animals.

### Mouse Model of Atopic Dermatitis

Eight-week-old mice were shaved with a clipper and a shaver, and 100 mg of Biostir AD (extract of HDM; Biostir Inc., Kobe, Japan) were applied onto the nape of neck on the next day. From then on, we applied the HDM extract twice per week for 6 weeks. Before each application of the HDM extract, re-grown hair was shaved, 150 μl of 4% sodium dodecyl sulfate solution were applied for barrier disruption, and mice were air-dried for 2–3 h before HDM application. Mice were euthanized at 14 weeks of age and multiple 4 μm sections from treated skin were obtained for histological analyses.

### Immunohistochemistry

#### For Paraffin-Embedded Sections

Slides were incubated with primary antibodies for 1 h at room temperature following rinsing with PBS. Antigen retrieval was performed for 10 min in TEG buffer. Slides were washed in 50 mM NH_4_Cl in PBS for 30 min and blocked by 1% BSA, 0.2% gelatine, 0.05% Saponin in PBS at room temperature for 10 min, three times. Primary antibody was diluted in 0.1% BSA, 0.3% Triton X-100 in PBS, overnight at 4°C. Antibodies against involucrin, loricrin, and filaggrin were purchased from Covance (Denver, PA). Rabbit polyclonal antibody PAR2 (H-99; sc-5597) was provided by Santa Cruz Biotechnology (Dallas, TX). Slides were rinsed three times for 10 min in PBS containing 0.1% BSA, 0.2% gelatine, and 0.05% saponin at room temperature and the secondary biotinylated antibody (goat-anti rabbit; Vector Labs, Burlingame, CA) was diluted in 0.1% BSA, 0.3% Triton X-100 in PBS. Elite Standard Vectastain ABC kit and DAB kit (both Vector Labs) were finally applied according to the manufacturer's instructions. Nuclei were counterstained with 4,6-diamidino-2-phenylindole (DAPI) (Dako, Glostrup, Denmark).

For hematoxylin and eosin (HE)-staining, paraffin-embedded sections of 4 μm were used. Microscopic analyses were performed using an Axioskop2 (Zeiss, Oberkochen, Germany) microscope and the Axiovision software Rel4.7 (Zeiss).

#### For Cryosections

Five-micrometer sections of frozen samples were fixed with methanol, followed by inactivation of endogenous peroxidase with 0.3% H_2_O_2_, blocking of endogenous biotin with Biotin-Blocking System (DAKO) and unspecific binding with 5% rabbit serum. The samples were incubated with the respective primary antibody, followed by incubation with the relevant horseradish peroxidase-labeled secondary antibody (Vector). Antibodies against CD11b (M1/70), CD3 (DaA3), CD4 (RM4-5), CD8 (Ssa1) were from Immunotools, Friesoythe, Germany; Gr1 (RB6–8C5) from BD, Heidelberg, Germany; ET-1 from Bachem, Torrance, CA; PGP9.5 from Abcam, Cambridge, MA. After incubation with streptavidin-peroxidase (Vector) and AEC+-Solution (Dako), samples were finally counterstained with hematoxylin (Dako).

#### NGF Staining

20 μm sections were incubated with a fluorescein isothiocyanate-conjugated rat anti-mouse NGF antibody (M-20; Santa Cruz).

#### LacZ Staining

LacZ expression was detected by incubating the tissue at 30°C overnight in 0.1% X-gal, 5 mM potassium ferricyanide, 5 mM potassium ferrocyanide, 1 mM magnesium chloride 0.002% NP-40, 0.01% sodium deoxycholate, PBS, pH 7.0. Finally, serum samples were used to determine total IgE by ELISA (eBioscience, San Diego, CA).

### Electron Microscopy

Skin samples were minced to <0.5 mm^3^ fragments, rinsed three times in 0.1 mol/l cacodylate buffer, and pre-fixed in half-strength Karnovsky's fixative, followed by postfixation in reduced 1% osmium tetroxide (OsO_4_) containing 1.5% ferrocyanide or in 0.2% ruthenium tetroxide (RuO_4_). Selected samples were immersed for 2 h in absolute pyridine for visualization of the cornified lipid envelope, followed by OsO_4_ postfixation, as described previously. The combination of osmium (OsO_4_) and ruthenium tetroxide (RuO_4_) postfixation protocols with pyridine pretreatment allowed us to assess the CE scaffold in relation to the extracellular lamellar bilayer system, as described previously. After staining with 2% aqueous uranyl acetate and embedding in Epon epoxy, ultrathin sections (600Å) were assessed using a Zeiss 10A electron microscope, operated at 60 kV.

### Assessment of Corneocyte Morphology

The number of SC layers was counted at ×3.5 to ×10 magnification. CE thickness was quantitated with an image analyzer, attached to the electron microscope camera, in the lowest SC layer (first SC layer above the SG–SC junction) vs. outermost SC layer by an unbiased observer who did 30 measurements taken from five images at ×125 magnification. The length of corneodesmosomes was measured between the first and second SC layer above the SG–SC junction at ×25 and expressed as corneodesmosome length/total CE length.

### Assessment of Permeability Pathways by Lanthanum Perfusion

Skin fragments prepared as described above were immersed in 4% lanthanum nitrate in 0.05 mol/l Tris buffer (pH 7.4) containing 2% glutaraldehyde and 1% paraformaldehyde for 1 h at room temperature. After lanthanum perfusion, the samples were washed and processed for electron microscopy, as described above.

### Lamellar Body Morphology, Secretion, and Extracellular Bilayer Structure

We assessed LB and the extent of LB secretion to determine whether Par2 knock-in interferes with secretion of LB contents. LB numbers were determined in granular cells two to three layers below the SG–SC junction by counting LBs at ×16 magnification using a calibrated grid. To assess the LB secretory system, the following criteria were assessed: (i) amount of accumulated lipid material at the SG–SC junction; (ii) presence of “entombed” LB within the corneocyte cytosol; and (iii) extent of extracellular delivery vs. corneocyte retention of a lipid hydrolase (acid lipase), which is concentrated in LB and normally secreted and segregated in toto within the SC interstices. For quantification of LB secretion, areas of secretion at the SG–SC junction were measured and correlated with the length of the bottom surface of the first SC layer on 10 random images at 16K magnification. Finally, on RuO_4_ postfixed tissue, the maturation and supramolecular organization of extracellular lamellar bilayer quantities were determined.

### Quantitative Real Time PCR (TaqMan®)

Skin biopsies and DRGs were homogenized in liquid nitrogen using a Mikro-Dismembrator U (Braun Biotech, San Diego, CA) and RNA was extracted with TRIzol reagent (Invitrogen, Carlsbad, CA). Samples from skin biopsies were tested with primers for murine PAR2, NGF, ETAR, and TSLPR. One microgram of RNA were reversed transcribed using SuperScript II (Invitrogen, Carlsbad, CA). Primers PAR2: forward, 5′-CCACGTCCGGGGATGCGAAG-3′; reverse, 5′-GTTGCGTCCCGGTGCAAGGT-3′; NGF: forward, 5′-TGATCGGCGTACAGGCAGA-3′; reverse, 5′-GAGGGCTGTGTCAAGGGAAT-3′; TSLPR: forward, 5′-CATCCGCGGGTGACCCCT-3′; reverse, 5′-TCCAGGGAAGGAGCCGCTGG-3′; ETAR: forward, 5′-GCTGGTTCCCTCTTCACTTAAGC-3′; reverse 5′-TCATGGTTGCCAGGTTAATGC-3′; GAPDH: forward, 5′-GCCTTCTCCATGGTGGTGAA-3′; reverse, 5′-GCACAGTCAAGGCCGAGAAT-3′. Twenty-five nanograms of cDNA were amplified per reaction, either in the presence of SYBR green master mix, or in the presence of TaqMan® universal master mix (Applied Biosystems, Foster City, CA). Gene-specific PCR products were measured by means of an ABI PRISM® 7000 Sequence Detection Systems (Applied Biosystems; stage 1, 50°C for 2 min, stage 2, 95°C for 10 min and stage 3, 95°C for 15 s, 60°C for 1 min, repeated 40 times). Gene expressions were related to the housekeeping gene and are presented as relative units of expression.

### Behavioral Tests

The fur on the rostral back was shaved and mice were habituated to the Plexiglas recording arena 1 week prior to testing. On the experiment day, animals were placed in an arena and videotaped for 30 min to assess spontaneous scratching. Following the recording, animals were tested with id injection of 10 μl of one of the following: vehicle (isotonic saline), histamine (Sigma-Aldrich, St. Louis, MO, 35 μg in saline), the PAR2/MrgprC11 agonist SLIGRL-NH2 (Quality Controlled Biochemicals, Hopkinton, MA, and GenScript, Piscataway, NJ; 35 μg in saline), or serotonin (Sigma, St. Louis, MO; 3 μg in saline). Intradermal (id) microinjections were made. Immediately following the id microinjection, mice were placed in the arena and videotaped for 30 min from above. Scratching elicited by each pruritogen subsided by the end of the 30-min recording period. Investigators left the room during videotaping. Videotapes were reviewed by investigators blinded to the treatment, and the number of scratch bouts was counted. A scratch bout was defined as one or more rapid back-and-forth hind paw motion(s) directed toward and contacting the injection site, and ending with licking or biting of the toes and/or placement of the hind paw on the floor. Hind paw movements directed away from the injection site (e.g., ear-scratching) and grooming movements were not counted. One-way ANOVA followed by the Bonferroni post-test or unpaired *t*-tests (two-tailed) was used to compare the total number of scratch bouts across pretreatment groups. In all cases *p* < 0.05 was considered to be significant.

Alloknesis was assessed as follows. At 5-min intervals, von Frey stimuli (bending force: 0.7 mN) were applied on the border of the lesional skin at 5 randomly selected sites. In pilot experiments we determined that application of von Frey stimuli within the lesional skin was ineffective. The presence or absence of a positive response, i.e., a hindlimb scratch bout directed to the site of mechanical stimulation, was noted for each stimulus before the next one was given. The alloknesis score was the total number of positive responses elicited by the three stimuli, i.e., 0, 1, 2, 3, 4, or 5. In one set of experiments, we tested the effect of the μ-opiate antagonist naltrexone on scratching and alloknesis. Naltrexone (1 mg/kg s.c., Dupont; Garden, NY) or saline was administered. In addition to this, we also performed subcutaneous injections with lidocaine (1%).

### Calcium Imaging

The animal was euthanized under sodium pentobarbital anesthesia, and upper- to mid-cervical DRGs were acutely dissected and enzymatically digested at 37°C for 10 min in HBSS (Invitrogen, Carlsbad, CA) containing 20 U/ml papain (Worthington Biochemical, Lakewood, NJ) and 6.7 mg/ml L-cysteine (Sigma), followed by 10 min at 37°C in HBSS containing 3 mg/ml collagenase (Worthington Biochemical). The ganglia were then mechanically triturated using fire-polished glass pipettes. DRG cells were pelleted; suspended in MEM with Earle's balanced salt solution (Gibco, Life Technologies, Carlsbad, CA) containing 100 U/ml penicillin, 100 μg/ml streptomycin (Gibco), 1× vitamin (Gibco), and 10% horse serum (Quad Five, Ryegate, MT); plated on poly-d-lysine-coated glass coverslips; and cultured for 16–24 h.

DRG cells were incubated in Ringer's solution (pH 7.4, 140 mM NaCl, 4 mM KCl, 2 mM CaCl2, 1 mM MgCl2, 10 mM HEPES, and 4.54 mM NaOH) with 10 μM Fura-2 AM and 0.05% of Pluronic F-127 (Invitrogen). Coverslips were mounted on a custom-made aluminum perfusion block and viewed through an inverted microscope (Nikon TS100, Technical Instruments, Burlingame, CA). Fluorescence was excited by UV light at 340 and 380 nm alternately, and emitted light was collected via a CoolSNAP camera attached to a Lambda LS lamp and a Lambda optical filter changer (Sutter Instrument, Novato, CA). Ratiometric measurements were made using Simple PCI software (Hamamatsu, Sewickley, PA) every 3 s.

Solutions were delivered by a solenoid-controlled eight-channel perfusion system (ValveLink, AutoMate Scientific, San Francisco, CA). One of the following agents was delivered for 30 s: histamine (100 μM), 5-HT (100 μM), and the PAR2/MrgprC11 agonist SLIGRL-NH2 (100 μM). Potassium chloride (144 mM) was always delivered at the end of each experiment. Ratios were normalized to baseline. Cells were judged to be sensitive if the ratio value increased by >10% of the resting level following chemical application. Only cells responsive to high potassium were included for analysis. Unpaired *t*-tests (two-tailed) were used to compare the mean Δpeak response (% of baseline) of DRG cells to the pruritogen across treatment groups. In all cases *p* < 0.05 was considered to be significant.

## Results

### Epidermal Par2 Overexpression Resulted in Spontaneous Eczema Formation and Intense Pruritus

**Figure 1:**
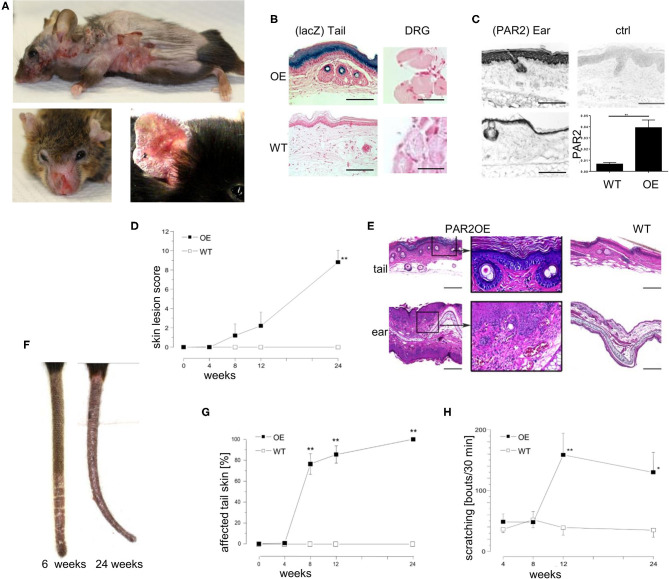
Par2 overexpression in the epidermis results in spontaneous eczema formation and intense pruritus. **(A)** Spontaneous phenotype of a representative PAR2OE mouse (shaved) at the age of 36 weeks before euthanasia. Please note that the eczematous skin lesions are mainly located at body sites which can be reached by scratching. **(B)** LacZ staining (light blue) on tail skin samples (left) and dorsal root ganglion neurons (DRG, right) of lesional PAR2OE and WT mice, counterstained with neutral red (pink) of the respective genotypes. Bars represent 300 μm (tail skin) and 40 μm (DRG). **(C)** PAR2 staining by immunohistochemistry on ear skin samples of lesional PAR2OE and WT mice. Upper right: polyclonal IgG control of lesional ear skin in PAR2OE. Bars represent 300 μm. Lower right: Par2 expression by qPCR in whole skin samples (analyzed relative to GAPDH expression). **(D)** Description of the spontaneous development of AD-like skin disease in PAR2OE and WT mice by a crude skin lesion score (0–12 points), details in methods; *n* = 7 per group. All graphs in this figure show mean ± SEM. *P < 0.05, **P < 0.01, (Mann–Whitney *U*-test). **(E)** HE stain of representative tail and ear skin samples including magnifications of lesional PAR2OE and WT mice at 14 weeks of age. Bars represent 500 μm. **(F)** Representative images of PAR2OE mouse tails at 6 and 24 weeks. **(G)** The graph depicts the extent of the skin lesions relative to total tail length (*n* = 7 per group). **(H)** Spontaneous scratching behavior of PAR2OE and WT mice during 30 min video recording; *n* = 7 per group.

We utilized *Grhl3*^Par2/+^ mice in which the mouse Par2 coding sequence, an internal ribosomal entry site and beta-galactosidase gene were inserted at the start codon of the grainyhead-like-3 (*Grhl3*) gene (*Grhl3*^PAR2/+^) by homologous recombination (6). *Grhl3*^Par2/+^ mice, hereafter called PAR2-overexpressing or PAR2OE mice, overexpressed PAR2, and expressed beta-galactosidase selectively in keratinocytes (Figure 1).

Although PAR2OE mice were born without any overt abnormalities, they started to develop eczematous skin lesions spontaneously after several weeks of life. This ultimately evolved to severe dermatitis with weight loss that necessitated euthanasia (Figure 1A). The skin lesions primarily developed at body sites that were accessible to scratching. We showed that the knock-in of Par2 is limited to the stratum spinosum and stratum granulosum of the epidermis (Figure 1B, as demonstrated by LacZ staining). Since dorsal root ganglion (DRG) neurons contain the cell bodies of sensory (afferent) neurons of the skin, and they will be studied in more detail later, we affirmed that DRG neurons did not stain positive for LacZ (Figure 1B). We did not perform additional experiments to exclude expression of the Par2-IRES-beta-galactosidase Grhl3 knockin. Although PAR2 was also expressed in the epidermis of WT littermates, skin lesions in PAR2OE mice showed a significantly higher PAR2 expression in the epidermis as assessed by immunohistochemistry and qPCR (about 8-fold higher expression, Figure 1C).

Using an arbitrary skin lesion score for dermatitis severity that evaluated “erythema/hemorrhage,” “edema,” “excoriation/erosion,” and “scaling/dryness” [maximum three points each (34)], we demonstrated the onset of visible skin alterations in PAR2OE mice from week 8 on, revealing a crescendo pattern toward later time points (Figure 1D). H&E staining of lesional skin of PAR2OE mice revealed histological characteristics of eczema such as spongiosis, parakeratosis, and a perivascular inflammatory infiltrate (Figure 1E). Skin lesions usually first appeared at the tip of the tail and spread proximally (Figures 1F,G) before appearing on the trunk.

The skin phenotype was accompanied by severe scratching behavior of the PAR2OE mice, with an ~3-fold increase in scratching bouts (Figure 1H). Overall, PAR2OE mice spontaneously developed severe scratching behavior and a skin phenotype grossly and histologically resembling eczema, characteristics typically found in human patients with atopic dermatitis (AD).

### House Dust Mite Extract Accelerated the Appearance of Eczematous Skin Lesions and Onset of Pruritus in PAR2OE Mice

**Figure 2:**
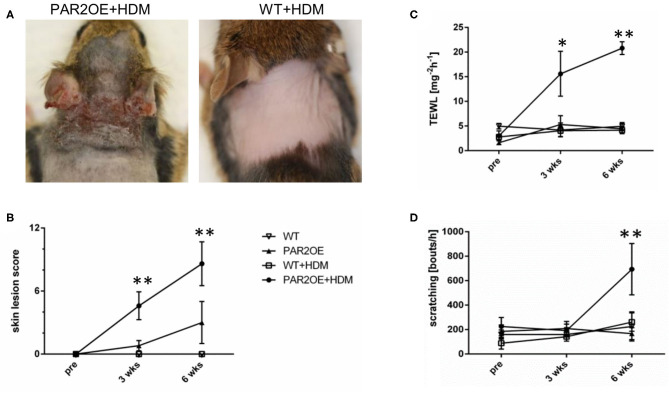
Application of house dust mites triggers eczematous skin lesions and pruritus in PAR2OE mice. **(A,B)** Skin phenotype by images (partially shaved mice) and skin lesion score of 14-week-old PAR2OE and WT mice, treated with HDM or vehicle for the last 6 weeks (*n* = 3–4 mice per group). All graphs in Figure 2 show mean ± SEM. *P < 0.05, **P < 0.01, (Mann–Whitney *U*-test). **(C,D)** As in panel **(B)**, 14-week-old PAR2OE and WT mice, treated with HDM or vehicle for the last 6 weeks, have been analyzed for transepidermal water loss (TEWL) and their spontaneous scratching behavior during 60 min video recording; *n* = 3–4 mice per group.

Since exposure to house dust mites (HDM) is associated with flare-ups of the skin disease in AD patients, and HDM allergens are known activators of PAR2, we investigated the effect of topical treatment with HDM extract (35, 36). PAR2OE and WT littermates were treated with HDM extract or vehicle ointment twice per week for 6 weeks, starting at 8 weeks of age. Before, 3 weeks after, and 6 weeks after the start of HDM treatment, skin lesion score, transepidermal water loss (TEWL), and scratching behavior were evaluated. Par2OE mice treated with HDM showed a marked increase in eczematous skin lesions compared to vehicle-treated PAR2OE mice, with average skin lesion scores of 8.6 ± 2.1 and 3.0 ± 2.0, respectively (Figures 2A,B). The low lesion score in the PAR2OE+vehicle group was expected and consistent with the spontaneous onset of skin lesions without any additional treatment (Figure 1D). WT littermates with and without HDM treatment did not show any skin lesions. Consistent with the skin lesion score, TEWL and spontaneous scratching bouts were also significantly elevated in the PAR2OE+HDM group compared to the other conditions examined (Figures 2C,D).

### PAR2OE Mice Displayed Skin Barrier Impairment Prior to Visible Skin Lesions

**Figure 3:**
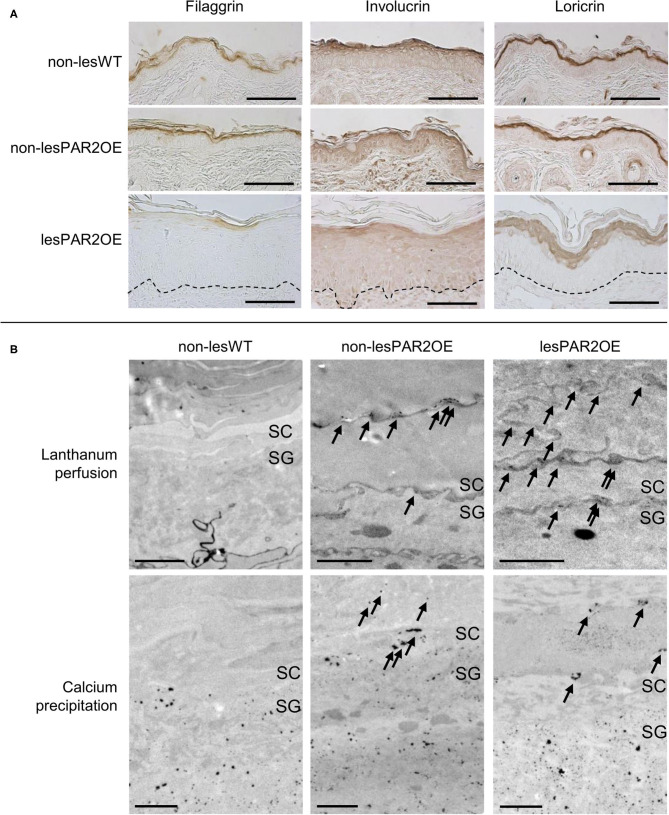
Structural impairment of barrier function in PAR2OE mice. **(A)** Immunohistochemical staining for filaggrin, involucrin, and loricrin in lesional and non-lesional skin of PAR2OE mice, as well as WT mice (representative of *n* = 4, age-matched 12-weeks-old mice). Manually drawn dashed line marks the basement membrane. Bars represent 100 μm. **(B)** Electron microscopy images are shown after lanthanum perfusion (upper row). Arrows mark intercellular lanthanum deposition in the SC in non-lesional and lesional PAR2OE skin. Calcium precipitation (lower row) was found in the SC in non-lesional PAR2OE and lesional PAR2OE skin (arrows). Bars represent 1 μm. SC, Stratum corneum; SG, Stratum granulosum.

For a deeper understanding of the pathophysiological processes in our PAR2OE mice, we first examined alterations of keratinocyte differentiation markers by immunohistochemistry (IHC), focusing on filaggrin, involucrin, and loricrin. IHC staining for these proteins revealed less signal in PAR2OE lesional skin compared to either WT skin or non-lesional PAR2OE skin (Figure 3A), suggesting decreased filaggrin, involucrin, and loricrin in PAR2OE skin lesions.

To functionally assess the epithelial barrier, we used the water-soluble tracer colloidal lanthanum for perfusion and visualization by transmission electron microscopy (TEM). Colloidal lanthanum is normally excluded from both the corneocyte cytosol and the extracellular matrix, and its perfusion stops in the stratum granulosum (SG). As expected, WT littermates showed this pattern of colloidal lanthanum staining. Interestingly, colloidal lanthanum was observed intercellularly in the stratum corneum (SC) of both lesional and non-lesional skin from PAR2OE mice (Figure 3B). We next analyzed the calcium gradient in a precipitation assay by TEM. In WT littermates, we observed a normal gradient with high calcium levels in the outer SG, and low or no calcium detection by this assay in the SC. In contrast, calcium leakage into SC was visible in both non-lesional and lesional skin of PAR2OE mice (Figure 3B), supporting our findings of impaired barrier function even in non-lesional skin of PAR2OE mice (Figure 3B).

**Figure 4:**
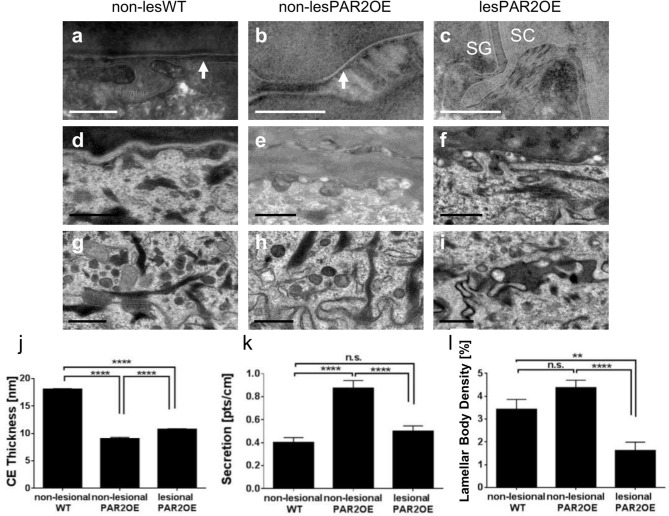
Morphological analyses of subcellular compartments and components by electron microscopy in lesional and non-lesional skin of PAR2OE mice, and WT mice. **(a–c,j)** Arrow shows cornified envelope (CE) and quantification is shown in panel **(j)** (*n* = 5). In each animal (age-matched 12-weeks-old mice), five pictures were analyzed and in four different locations CE thickness was measured (arrows point toward CE). Bars represent 0.2 μm. SC, Stratum corneum; SG, Stratum granulosum. **(d–f,k)** Lamellar body secretion. Quantification of secreted lamellar bodies is shown in panel **(k)**, in each animal 10 pictures were analyzed (*n* = 10). Bars represent 0.5 μm. **(g–i,l)** Lamellar body density. Quantification (*n* = 10) is shown in **(l)**. In each animal 10 pictures were taken and in each picture, two different areas were counted (when counting, three rotations of counting instrument, resulting in 60 area analyses). Bars represent 0.5 μm. **(j–l)** Graphs show mean ± SEM. **p < 0.01, ****p < 0.0001, n.s., not significant (unpaired Student's *t-*test).

Detailed morphological analyses of the epidermis of lesional PAR2OE skin by TEM revealed an abundance of abnormalities, thus we focus here on alterations in non-lesional PAR2OE skin (Figure 4). Most notably, non-lesional PAR2OE skin displayed a regular but significantly thinner cornified envelope (Figures 4a–c,j) and a significant increase in lipid secretion (Figures 4d–f,k) compared to WT skin. The desmosomes in PAR2OE epidermis were shorter in SG and in SC-SG junction, and SC extracellular lamellar bilayers were also observed to be abnormal (and non-existent in ruthenium postfixed samples; data not shown). Lamellar body density was normal in non-lesional but reduced in lesional PAR2OE epidermis (Figures 4g–i,l), while keratin bundles, and mitochondria morphology and number were normal independent of lesion development (data not shown).

### HDM-Treated PAR2OE Mice Displayed Immunological Characteristics of AD

**Figure 5:**
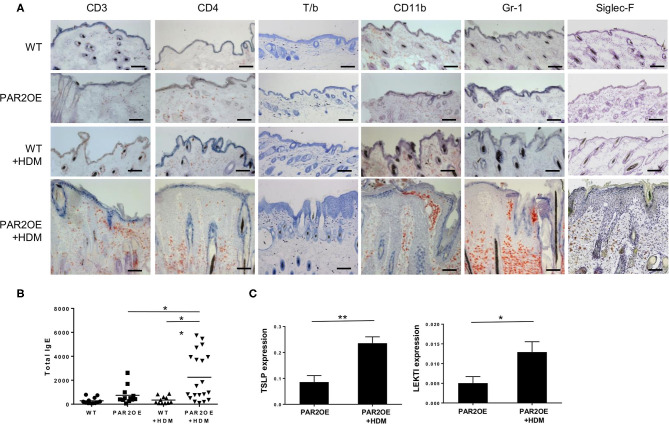
The eczematous skin lesions in HDM-treated PAR2OE mice display immunological characteristics of atopic dermatitis. All samples derive from 14-week-old PAR2OE and WT mice, treated with HDM or vehicle for the last 6 weeks. **(A)** Representative toluidine blue (T/b), CD3, CD4, CD11b, Gr-1, Siglec-F immunohistochemistry stainings. Bars represent 200 μm. **(B)** Total-IgE in the blood of 14-week-old PAR2OE and WT mice, after 6 weeks of HDM or vehicle treatment. Bars represent the mean (*n* = 12–20 mice). **(C)** Expression of TSLP and LEKTI by qPCR in whole skin samples (*n* = 5 mice per group). *p < 0.05, **p < 0.01, (unpaired Student's *t-*test).

To further characterize our mouse model, we next assessed the infiltration of inflammatory cells in PAR2OE and littermate mouse skin with and without HDM treatment (as in Figure 2B). Dermal mast cells were increased in PAR2OE+HDM compared to all other groups (Figure 5A). By immunohistochemistry infiltrating lymphocytes were found to be mainly CD3^+^ and CD4^+^ (Figure 5A); no CD8^+^ T cells were found in any group (data not shown). CD11b^+^ and Gr-1^+^ cells (monocytes and granulocytes) and Siglec-F^+^ cells (eosinophils) were also significantly elevated in PAR2OE+HDM mice. Interestingly, total IgE levels in the blood were also elevated in PAR2OE+HDM mice (Figure 5B). Since TSLP has been identified as an important link in PAR2-mediated itch (37), we analyzed TSLP and LEKTI in the skin samples and found significantly elevated levels for both compounds in PAR2OE after HDM treatment (Figure 5C).

### Evidence for Enhanced Epidermo-Neuronal Communication in Skin and Dorsal Root Ganglion Neurons in PAR2OE Mice

**Figure 6:**
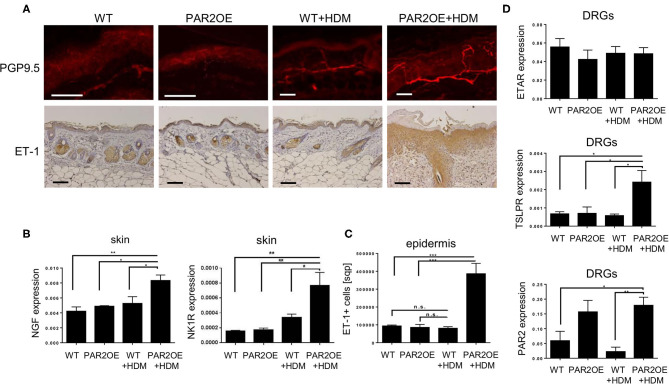
Evidence for increased epidermo-neuronal communication, and Par2 upregulation on dorsal root ganglion neurons. All samples derive from 14-week-old PAR2OE and WT mice, treated with HDM or vehicle for the last 6 weeks. **(A)** Representative immunofluorescence of the ear (PGP9.5) and immunohistochemistry of the neck skin (ET-1) images of the skin. ET-1 staining is quantified in panel **(C)**. All bars represent 100 μm. **(B)** Expression of NGF by qPCR in whole skin samples (*n* = 4–5 mice per group). All graphs in this figure show mean ± SEM. *p < 0.05, **p < 0.01, ***p < 0.001 (unpaired Student's *t-*test). **(C)** Epidermal ET-1 staining by immunohistochemistry were automatically quantified in squarepixels (sqp) using ImageJ (*n* = 4–5 mice per group, three slides per mouse). In addition, mean brown intensity is also increased in PAR2OE+HDM compared to PAR2OE (*p* = 0.015, data not shown). **(D)** Expression of ETAR, TSLPR, and Par2 by qPCR on dorsal root ganglion (DRG) neurons.

Due to the significant scratching behavior in PAR2OE mice (Figure 1H), we addressed nerve anatomy and neuro-epidermal communication. Increased prevalence of PGP9.5-positive neurons and NGF mRNA both suggested an increase in nerve fiber density in HDM-treated PAR2OE mice compared to WT littermates and PAR2OE without HDM-treatment (Figures 6A,B). The potent pruritogen endothelin-1 (ET-1) is implicated in histamine-independent pruritus in mice and humans, especially in skin diseases with increased pruritus such as AD and prurigo nodularis, in which antihistamines are hardly effective (38). Staining for epidermal ET-1 was significantly more widespread and pronounced in the epidermis of PAR2OE+HDM mice relative to all other groups (*p* = 0.0025, Figure 6C), while expression of endothelin A receptor (ETAR) persisted on the dorsal root ganglion (DRG) neurons (Figure 6D). Expression of TSLPR, the receptor for the epidermal cytokine TSLP, was significantly elevated in PAR2OE+HDM DRG cells (*n* = 4–5 mice per group). Of note, Par2 expression was significantly elevated in DRG neurons of PAR2OE mice, independent of treatment with HDM. No beta-galactosidase staining was detected in DRG cells (Figure 1B), suggesting that increased Par2 expression in DRG cells reflects increased expression from the endogenous *F2rl1* locus rather than expression of the Par2-IRES-beta-galactosidase *Grhl3* knockin.

**Figure 7:**
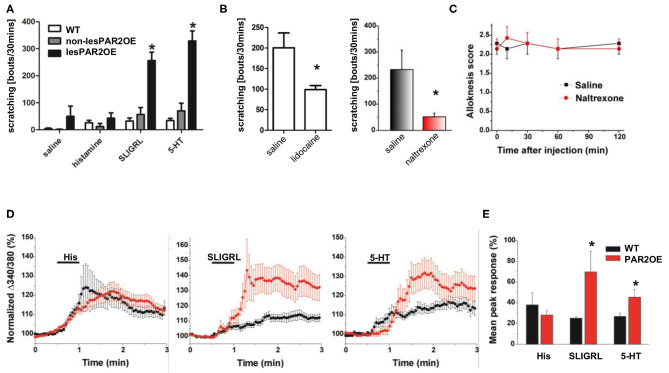
Analysis of sensitization of itch signaling pathways in PAR2OE mice. **(A)** Injection of saline, histamine, SLIGRL, and 5-HT into lesional and non-lesional PAR2OE mice, as well as WT mice. Spontaneous scratching bouts have been substracted from the results (*n* = 3–7 for each group). All graphs in this figure show mean ± SEM. *significantly different compared to WT mice or non-lesional PAR2OE mice (*p* < 0.05, one-way ANOVA followed by the Bonferroni test). **(B)** Subcutaneous application of lidocaine (Lido) or naltrexone (Nal) to PAR2OE mice, each in comparison to saline (Sal) application. Determination of spontaneous scratching behavior during 30 min video recording (*n* = 8 mice per group). Spontaneous scratching was not substracted here as in panel **(A)**. *p < 0.05 (unpaired *t*-test). **(C)** Alloknesis score over 120 min after injection of naltrexone vs. saline (*n* = 7 mice per group). WT mice had alloknesis scores of 0 (data not shown). **(D)** Calcium responses of cultured DRG cells from PAR2OE and littermate WT mice after stimulation with histamine, SLIGRL, and 5-HT [same pruritogens as injected *in vivo* in panel **(A)**]. **(E)** Summary of the results from panel **(D)**: mean Δpeak response is depicted here. *p < 0.05 (unpaired *t*-test).

To address the functional consequences of increased nerve fiber density and increased Par2 expression on DRG neurons, we injected the Par2/MrgprC11 agonist peptide (SLIGRL) as well as two independent pruritogens, histamine and serotonin (5-HT). Despite ubiquitous Par2 overexpression in DRG neurons of PAR2OE mice, injection of SLIGRL only resulted in significantly increased scratching bouts (hyperknesis) in lesional PAR2OE (lesPAR2OE) relative to WT littermates (Figure 7A). Consistent with the increase in nerve fiber density, injection of 5-HT into lesPAR2OE also elicited enhanced scratching similar to that evoked by SLIGRL, while histamine did not. We further analyzed the scratching behavior after PNS blockade by lidocaine and naltrexone. Both compounds were able to significantly reduce the spontaneous scratching in PAR2OE mice (Figure 7B).

PAR2OE mice exhibited a significant increase in touch-evoked scratching (alloknesis score ~2) compared to WT animals, which exhibited no alloknesis (alloknesis score = 0). Figure 7C shows that alloknesis was not affected by naltrexone, as the alloknesis score was equivalent following naltrexone or control saline injections.

To further investigate the basis of scratching behavior, we used calcium imaging to investigate pruritogen-evoked responses in DRG cells from PAR2OE and WT littermates. Mean calcium responses following stimulation with the pruritogens histamine, SLIGRL, and 5-HT are shown in Figure 7D and summarized in Figure 7E. Calcium responses to SLIGRL and 5-HT but not histamine were enhanced in DRG cells from PAR2OE mice, consistent with scratching response to these agents (Figure 7A). Thus, increased nerve fiber density and hypersensitivity, potentially secondary to receptor overexpression, may contribute to increased scratching behavior of the PAR2OE mice.

## Discussion

The cellular circuits that link skin epithelium, immune cells and the skin nervous system in AD are very poorly understood. Here, we present *in vitro, in vivo*, and *ex vivo* data on spontaneous and house dust mite (HDM)-triggered development of AD-like skin disease in mice with epidermal overexpression of PAR2 (PAR2OE). The hallmarks of this dermatitis comprise severe pruritus, characteristic inflammatory infiltrate, increased IgE levels, as well as barrier dysfunction. Our findings demonstrate that PAR2, activated by exogenous and/or endogenous proteases, can contribute to processes that elicit all hallmarks of AD.

Our findings of spontaneous development of AD-like skin disease in PAR2OE mice fit well with what is currently known about barrier dysfunction in AD pathogenesis. SPINK5 knockout mice (Spink5^−/−^) have been used to investigate the lack of one key serine protease inhibitor LEKTI, which models the human disease Netherton syndrome. Loss of LEKTI leads to hyperactivity of epidermal proteases, followed by stratum corneum detachment, resulting in enhanced allergen absorption and formation of acanthosis, papillomatosis, parakeratosis, and influx of immune cells (22, 39).

KLK5 activity is deregulated upon loss of LEKTI. KLK5 can activate PAR2, and PAR2 contributes to TSLP overexpression in LEKTI deficient mouse skin (39). Thus, since PAR2 deficiency reduces inflammation without preventing the major skin pathology associated with LEKTI deficiency, we predicted PAR2OE mice to display a less severe phenotype that was more inflammatory of nature. Since *Spink5*^−/−^ mice die from dehydration a few hours after birth, PAR2OE mice could constitute a more suitable model for AD-like skin disease and potentially also the “atopic march” (16). The term “atopic march” describes the observation that people with atopic dermatitis are more likely to develop food allergies, allergic asthma, and allergic rhinitis subsequently.

PAR2 activation has been shown to induce TSLP in keratinocytes, which is considered a key trigger in the initiation and maintenance of AD and the “atopic march” (40, 41). Consistent with the ability of keratinocyte-derived TSLP to activate neurons to induce itch (37), we observed increased levels of TSLP in the skin of HDM-treated PAR2OE mice with significantly increased spontaneous scratching. Eczema-like skin lesions developed primarily at body sites accessible to scratching, underlining the clinical importance of the itch-scratch-cycle in eczema pathogenesis (23). With their slow but consistent development of pruritus and eczema, PAR2OE mice mimic the clinical course of AD in children closer than other mouse models of AD (e.g., flaky tail mouse, SPINK5^−/−^, or filaggrin^−/−^) (39, 42, 43). In addition, topical HDM treatment triggers or aggravates the skin disease in PAR2OE mice, which is also a common feature in AD patients. It has already been shown that PAR2 activation plays a central role during HDM sensitization, and Par2-deficient mice display significantly reduced type 2 immunity-related inflammation during HDM challenge (28, 36, 44, 45).

With regard to skin barrier function, WT skin and non-lesional PAR2OE skin revealed no significant differences in filaggrin, involucrin, and loricrin levels, but lesional PAR2OE skin showed a remarkable decrease in the level of all three epidermal proteins. Interestingly, even non-lesional skin of PAR2OE mice revealed a loss of the epidermal calcium gradient and a leaky epidermal water barrier. By morphological analysis of non-lesional PAR2OE skin, we found a regular but thinner cornified envelope, increased lipid secretion, and abnormal SC extracellular lamellar bilayers in comparison to WT mice. The response to barrier damage includes secretion of preformed lamellar bodies, followed by increased lipid synthesis, further production/secretion of new lamellar bodies, and organization of secreted and processed lipids into mature lamellar membrane structures, thereby restoring barrier function (14, 46). Our findings of an impaired skin barrier in PAR2OE mice with reduced CE thickness and secondary increases in lipid secretion are consistent with earlier descriptions of skin barrier defects induced by excess KLK-induced activation of PAR2, and its restoration by Par2 deficiency (39, 47, 48).

Analyses of the inflammatory infiltrate in HDM-treated PAR2OE mice revealed an increase of CD4+ helper T cells, but not of CD8+ effector T cells. Mast cells and eosinophilic granulocytes were also significantly elevated in HDM-treated PAR2OE mice. These effects were absent in HDM-treated littermates, vehicle-treated PAR2OE, and WT mice. Increased total IgE levels were observable, although not all HDM-treated PAR2OE mice displayed an identical increase in IgE levels. The same immune cell subpopulations were described to be elevated in *Spink5*^−/−^ skin, but the activation of the immune system started during embryonic development and was significantly more pronounced on embryonic day 19 (4-fold increase in mast cells, 6-fold increase in eosinophils) than in 14-week-old HDM-treated PAR2OE mice (15, 39).

Due to the severe scratching phenotype in PAR2OE mice especially after HDM treatment, we investigated possible links between altered keratinocyte signaling and sensory dorsal root ganglion (DRG) neurons. In skin lesions of HDM-treated PAR2OE mice, we observed increased nerve fiber density and an increased expression of epidermal ET-1, which we recently described as a potent inducer of histamine-independent pruritus in chronic itch (38). Secondly, we observed upregulation of TSLPR on DRG neurons, which in the context of Par2-mediated TSLP expression suggests the involvement of an additional pruritogenic pathway regulated by Par2 (37). Thirdly, the influx and polarization of immune cells results in secretion of pro-inflammatory cytokines that activate DRG cells directly; for example, IL-31 from T_H_2 cells may act on sensory neurons in the generation of T cell-mediated itch in AD (49). Our hypothesis of enhanced neuroimmune signaling in lesional PAR2OE skin is supported by our finding that injection of the Par2 agonist SLIGRL resulted in enhanced scratching behavior (hyperknesis). This finding was confirmed by demonstrating enhanced SLIGRL-evoked calcium responses in DRG cells from PAR2OE mice. Interestingly, 5-HT, but not histamine, also elicited enhanced scratching and responses in DRG cells, suggesting differential sensitization of non-histaminergic pruriceptors in PAR2OE mice, similar to previous studies using models of dry skin pruritus (50), atopic dermatitis (51), or contact hypersensitivity (52). The increased density of neuronal afferents we found in PAR2OE lesional skin may also contribute.

In summary, we present a body of evidence using dermatological, behavioral, neuroscientific, and immunological approaches indicating that increased epidermal Par2 activity is sufficient to drive many features of human AD in a mouse model. Additionally, increased epidermal Par2 activity facilitates skin sensitization to exposure to HDM extract and pruritogens, another feature of human AD. The remarkable effects of HDM extract in this model may be due to direct activation of keratinocyte PAR2 by PAR2-activating proteases known to be present in HDM extracts and/or to other effects of HDM enabled and enhanced by the PAR2-driven barrier defect. This complex project did not investigate protease content and level changes in the skin mediated by Par2 knock-in, in different ages of the mice, different eczema stages, and different topical treatments initially and over several weeks (e.g., by HDM, SDS, or petrolatum). While sustained barrier defects (regardless of cause) stimulate pro-inflammatory immune cascades (14) and can be the primary cause of inflammation in AD patients, the same is true for dysregulated epithelial and immune cell functions, which lead to pruritus, attraction of inflammatory immune cells, and a secondary disruption of the barrier due to this cellular influx and its mediators (“inside-out theory”) (53–56). Our studies provide strong evidence that signaling processes in keratinocytes can serve a primary role.

Our results suggest that Par2 signaling in keratinocytes triggers epidermal responses that are sufficient to trigger neuronal sensory and inflammatory responses in our AD model. Studies of global Par2 deficiency suggests that Par2 can play a necessary role in related models. PAR2OE mice displayed striking parallels to human AD: (i) no overt skin disease at birth, (ii) slowly crescendo-type development of spontaneous skin lesions, in association with significant pruritus, (iii) aggravation of the skin disease including pruritus upon topical exposure to HDM, (iv) an initially intact skin barrier that becomes dysfunctional over time and seems to precede the skin lesions, (v) dermal infiltration of characteristic immune cells and increased IgE production, (vi) involvement of different pruritogenic pathways resulting in direct and indirect activation of skin-innervating DRG cells, (vii) secondary upregulation of neuronal Par2 on DRG neurons during chronification of the AD lesions in PAR2OE, and (viii) increased sensitivity to various pruritogens. These and other data raise the question of whether the keratinocyte-protease-PAR2 system may mediate barrier defects, sensory signaling and neuro-immune communication in human AD, and whether PAR2 antagonists and/or selective protease inhibitors may be a novel approach for its treatment. This initial description of pathophysiological processes in PAR2OE mice warrants further in-depth analysis of the different mechanisms involved (e.g., on triggers of the skin barrier defect and its time course, on more details of the infiltrating immune cells, their activation, mediators, and regulation, etc.) to better understand its fidelity as a model for human AD and its potential utility for evaluation of candidate therapeutic approaches.

## Data Availability Statement

The datasets generated for this study are available on request to the corresponding author.

## Ethics Statement

The animal study was reviewed and approved by UCSF and UCD-Institutional Animal Care and Use Committee and conducted in accordance with the National Institutes of Health Guide for Care and Use of Laboratory Animals.

## Author Contributions

TB, AI, SR, and MSt designed the research. TB, AI, CK, FC, MSu, and JB performed experiments for Figures 1, 2, 5, 6. TB, MSu, DC, and PE performed experiments for Figures 3, 4. Experiments for Figure 7 were performed by TA and ECar. TB, AI, and MSt drafted the manuscript. All authors discussed data and worked on the mansucript for finalization.

## Conflict of Interest

The authors declare that the research was conducted in the absence of any commercial or financial relationships that could be construed as a potential conflict of interest.

## Abbreviations

AD: atopic dermatitis
CE: cornified envelope
DRG: dorsal root ganglion
HDM: house dust mite
HS: healthy skin
IHC: immunohistochemistry
IFN: interferon
IL: interleukin
LB: lamellar body
lesPAR2OE: lesional skin of PAR2OE
PAR2: protease-activated receptor 2
PAR2OE: PAR2 overexpression
SC: stratum corneum
SG: stratum granulosum
TEM: transmission electron microscopy
Th1: T helper cell type 1
Th2: T helper cell type 2
Th17: T helper cell type 17
Th22: T helper cell type 22
TNF: tumor necrosis factor
TSLP: thymic stromal lymphopoietin.
